# Evidence for liver energy metabolism programming in offspring subjected to intrauterine undernutrition during midgestation

**DOI:** 10.1186/s12986-019-0346-7

**Published:** 2019-03-18

**Authors:** Xiaoling Zhou, Hong Yang, Qiongxian Yan, Ao Ren, Zhiwei Kong, Shaoxun Tang, Xuefeng Han, Zhiliang Tan, Abdelfattah Z. M. Salem

**Affiliations:** 1 CAS Key Laboratory for Agro-Ecological Processes in Subtropical Regions, National Engineering Laboratory for Pollution Control and Waste Utilization in Livestock and Poultry Production, South-Central Experimental Station of Animal Nutrition and Feed Science in the Ministry of Agriculture, Institute of Subtropical Agriculture, The Chinese Academy of Sciences, Yuanda 2nd Road 644#, Furong District, Changsha, P.O. Box 10#, Hunan 410125 People’s Republic of China; 2 University of Chinese Academy of Science, Beijing, 100049 China; 3 College of Animal Science, Tarim University, Alaer, 843300 China; 4 Hunan Co-Innovation Center for Utilization of Botanical Functional Ingredients, Changsha, 410128 China; 5 Hunan Co-Innovation Center of Animal Production Safety, CICAPS, Changsha, 410128 China; 6 Facultad de Medicina Veterinaria y Zootecnia, Universidad Autónoma del Estado de México, Tlaphan, Mexico

**Keywords:** Intrauterine undernutrition, Hepatic energy metabolism, Metabolic profiling, Gluconeogenesis, Circadian rhythm, Metabolic programming

## Abstract

**Background:**

Maternal undernutrition programs fetal energy homeostasis and increases the risk of metabolic disorders later in life. This study aimed to identify the signs of hepatic metabolic programming in utero and during the juvenile phase after intrauterine undernutrition during midgestation.

**Methods:**

Fifty-three pregnant goats were assigned to the control (100% of the maintenance requirement) or restricted (60% of the maintenance requirement from day 45 to day 100 of midgestation and realimentation thereafter) group to compare hepatic energy metabolism in the fetuses (day 100 of gestation) and kids (postnatal day 90).

**Results:**

Undernutrition increased the glucagon concentration and hepatic hexokinase activity, decreased the body weight, liver weight and hepatic expression of *G6PC*, *G6PD*, and *PGC1α* mRNAs, and tended to decrease the hepatic glycogen content and *ACOX1* mRNA level in the dams. Maternal undernutrition decreased the growth hormone (GH) and triglyceride concentrations, tended to decrease the body weight and hepatic hexokinase activity, increased the hepatic *PCK1*, *PCK2* and *PRKAA2* mRNAs levels and glucose-6-phosphatase activity, and tended to increase the hepatic *PRKAB1* and *CPT1α* mRNAs levels in the male fetuses. In the restricted female fetuses, the hepatic hexokinase activity and *G6PC* mRNA level tended to be increased, but *PKB1* mRNA expression was decreased and the *ACACA*, *CPT1α*, *NR1H3* and *STK11* mRNA levels tended to be decreased. Maternal undernutrition changed the hepatic metabolic profile and affected the metabolic pathway involved in amino acid, glycerophospholipid, bile acid, purine, and saccharide metabolism in the fetuses, but not the kids. Additionally, maternal undernutrition increased the concentrations of GH and cortisol, elevated the hepatic glucose-6-phosphate dehydrogenase activity, and tended to decrease the hepatic glycogen content in the male kids. No alterations in these variables were observed in the female kids.

**Conclusions:**

Maternal undernutrition affects the metabolic status in a sex- and stage-specific manner by changing the metabolic profile, expression of genes involved in glucose homeostasis and enzyme activities in the liver of the fetuses. The changes in the hormone levels in the male fetuses and kids, but not the female offspring, represent a potential sign of metabolic programming.

## Background

Maternal undernutrition is a concerning problem for human health [1] and animal husbandry production [2]. Stress caused by nutrient deficiency in utero often induces metabolic disturbances for adjustment to the nutrient-poor environment [3, 4]. These metabolic disturbances are programmed in the fetus [5–7], altering the preset metabolic routine and increasing the risk of metabolic disorders. Epidemiological investigations have confirmed the association between uterine undernutrition and aberrant metabolism, and clinical symptoms of metabolic disorders often occur progressively in adults after long-term malnourishment [3, 7].

Metabolic disorders are primarily mediated by disruption of energy homeostasis. The liver is the largest parenchymatous organ that modulates systemic energy homeostasis in mammals [8] and is the first sensor and processor to deliver maternal nutrients to a growing fetus via the umbilical vein [9]. The capacity of the liver to achieve and maintain its normal function and functional reserve is established early in life [10]. During intrauterine development, the liver initially functions as the principal hematopoietic organ for the first eight weeks; the functional shift from hematopoiesis to hepatogenesis begins during midgestation. During this period, the hepatoblast population proliferates and expands dozens of times to define the basic volume of the liver, and differentiated cells develop morphologically and metabolically transform into parenchymal hepatocytes [11]. Differentiating hepatocytes are sensitive to the intrauterine microenvironment, including nutrient status, during midgestation [12]. At birth, the neonatal liver remains relatively immature. The functional capacity of liver tissue undergoes several changes during the early postnatal period, and the liver is basically mature at the juvenile stage [13]. The juvenile stage is one of the critical periods for examining potential intrauterine programming in the liver, and preventive measures should be implemented during this stage before the possible onset of an adult metabolic abnormity.

During midgestation, maternal undernutrition alters the liver weight of fetuses in sheep [7, 14–16] and cattle [17], affects insulin secretion [7], hepatic gene expression and epigenetic modification of gluconeogenic enzyme in sheep [16], and changes the fetal liver metabolite profile in baboons [18]. These studies partly reveal the effects on liver function in fetuses or in adult offspring. However, the mechanism by which this disturbance develops during the early postnatal stage has not been elucidated. We hypothesized that more signs of a programmed metabolic disturbance could be traced in the livers of juveniles. Small ruminants are a long-standing animal model used to study metabolic disorders caused by maternal nutrient restriction because of their roles in husbandry production and their similarity to humans in terms of fetal weight and of organ development and maturity at birth [19]. According to previous studies [20, 21], 40% maternal energy restriction leads to fetal programming of the liver weight and insulin concentration in goats. Thus, using a goat model of maternal undernutrition during midgestation, the effects of maternal undernutrition during midgestation on liver energy metabolism in pregnant goats and their offspring at the level of circulating blood, hepatic metabolites, genes and enzymes were examined. This information will expand our knowledge of metabolic disorders during ontogeny and may help predict metabolic diseases later in life.

## Methods

### Ethical approval

All the protocols used in this study were approved by the Animal Care Committee according to the Animal Care and the Use Guidelines of the Institute of Subtropical Agriculture, Chinese Academy of Sciences, Changsha, China (No. KYNEAAM-2015-0009).

### Experimental design and animal management

**Fig. 1:**
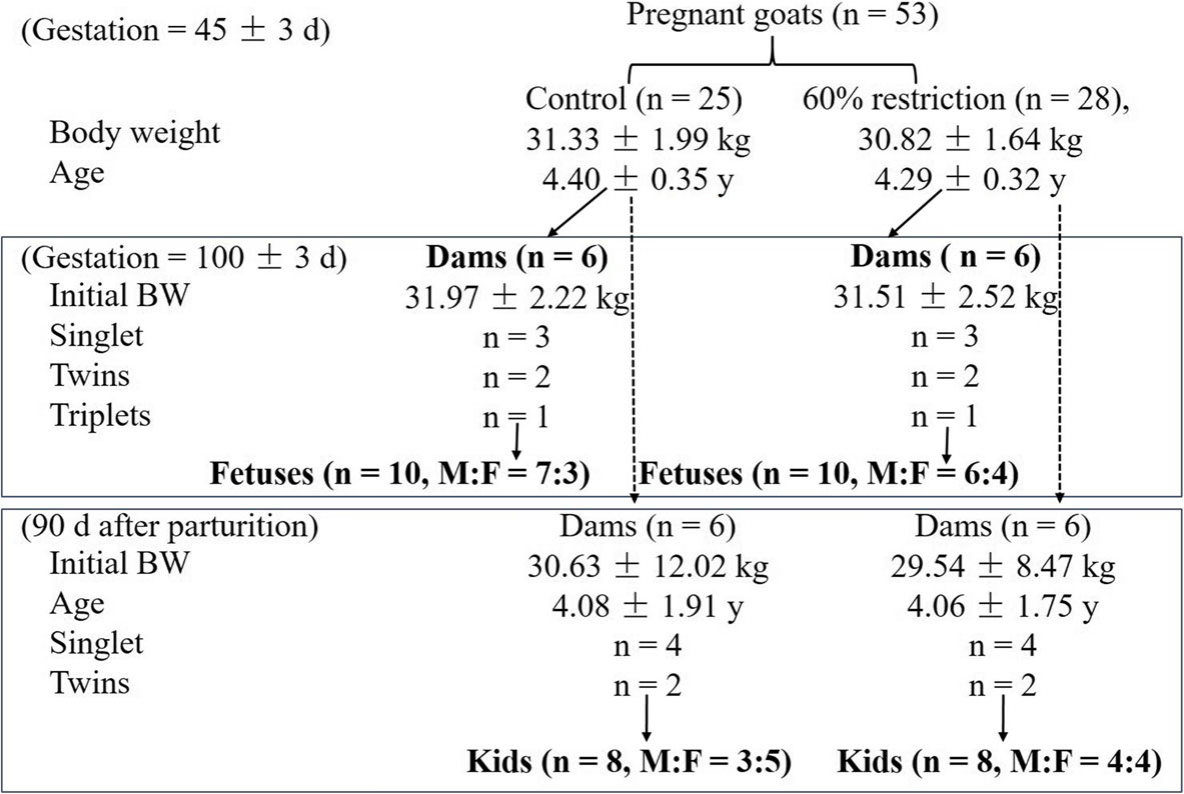
Experimental design. Values are presented as the mean ± SE

Fifty-three goats (45 ± 3 d of gestation, *Liuyang* black goat, local meat breed) were selected according to body weight (BW) and age and then randomly assigned to the control [C, 100% of the maintenance requirements suggested in the feeding standard of meat-producing sheep and goats of China (2004)] or restricted (R, 60% of the maintenance requirements) group, as illustrated in Fig. 1. All dams were housed in individual pens and fed twice (08:00 and 16:00) per day with a 50:50 ratio of concentrate to roughage, with free access to drinking water. The ingredients of the experimental diet on a dry matter basis were 50% fresh-mowed *Miscanthus*, 33.5% maize, 10.33% soybean meal, 4.0% fat power, 0.49% calcium carbonate, 0.46% calcium bicarbonate, 0.22% sodium chloride, and 1.0% premix, with 119 g of MgSO_4_•H_2_O, 2.5 g of FeSO_4_•7H_2_O, 0.8 g of CuSO_4_•5H_2_O, 3 g of MnSO_4_•H_2_O, 5 g of ZnSO_4_•H_2_O, 10 mg of Na_2_SeO_3_, 40 mg of KI, 30 mg of CoCl_2_•6H_2_O, 95,000 IU of vitamin A, 17,500 IU of vitamin D, and 18,000 IU of vitamin E in each kilogram of premix. This diet contained 11.78 MJ/kg metabolic energy, 12.05% crude protein, 28.32% acid detergent fiber, 0.53% calcium and 0.2% phosphorus. The restricted feeding in the R group was conducted by providing 60% of the feed allowance of the C group from 45 to 100 d of gestation, and the actual restriction level (1.04 kg/d for each dam in the C group vs. 0.62 kg/d for each dam in the R group) was 60.2% after measuring the daily feed allowance and refusals. Four dams from each group aborted during this period. At day 100 of gestation, the litter size of each dam was examined using portable ultrasonography (Aloka SSD-500 with a 5-MHz linear probe Aloka, Shanghai, China). Then, six pregnant goats from each group were selected for harvest to obtain a similar initial BW and equal litter size. Ten fetuses from these six dams were obtained from each group. The detailed experimental design and inclusion criterion for the subjects are shown in Fig. 1.

At day 101 of gestation, the feed restriction was lifted, and the remaining dams were fed 100% of the requirement and were managed as described above during the following experimental period. After parturition, neonatal kids were nursed by their dams until preweaning at day 50 after birth, but two litters from the C group and four litters from the R group died during this period. Between days 50 and 60 after birth, preweaning was conducted by separating the offspring from their dams from 0800 to 1600 h during the daytime, and a mixed diet of starter and fresh *Miscanthus* spp. was provided with a 20:80 ratio of roughage to concentrate during this period. After complete weaning at day 60, all kids in each group were housed together and provided ad libitum access to drinking water and the above diet of starter and fresh *Miscanthus* spp. (20:80) twice daily (0800 and 1600). The average dry matter intake of the kids in the C and R groups from day 61 to day 90 was 0.31 and 0.29 kg/d, respectively. The ingredients of the kid diet were 20% fresh-mowed *Miscanthus*, 36% maize, 14.4% wheat bran, 14.16% soybean meal, 6.4% whey power, 6.4% fat power, 0.24% calcium carbonate, 0.8% calcium bicarbonate, 0.4% sodium chloride, and 1.2% premix, with a composition identical to the composition of the premix provided to the dams. The kid diet contained 15.19 MJ/kg metabolic energy, 15.52% crude protein, 11.67% acid detergent fiber, 0.76% calcium and 0.32% phosphorus. At 90 d of age, after the initial BW, the age and litter size of the dams in each group were matched (presented in Fig. 1), eight eligible kids from each group were selected for harvest, and the mismatched offspring were discharged from the study.

### BW measurement and blood and liver tissue sampling

The BWs of dams at day 100 of gestation and postnatal kids at day 90 were measured before the morning feeding. Feed was withdrawn for 24 h, and fresh water was offered ad libitum. Then, blood was collected from the jugular veins of dams and kids after electronarcosis. Following exsanguination and ventrotomy of the dams, fetal blood samples were collected from the umbilical cord. Plasma was separated by centrifugation at 1000×*g* for 10 min at 4 °C and stored at − 80 °C for subsequent analysis. Immediately after the livers of all animals were weighed and washed with 0.09% sterile physiological saline, slices of tissue samples were snap-frozen in liquid nitrogen and then stored at − 80 °C until further analysis.

### Measurement of blood biochemical and hormonal parameters

Plasma samples were thawed at 4 °C, and the concentrations of growth hormone (GH), insulin, insulin-like growth factor 1 (IGF-I), insulin-like growth factor 2 (IGF-II), and cortisol were measured according to the manufacturer’s instructions (Cusabio Biotech Company Limited, Wuhan, China). Glucagon concentrations were also measured (Nanjing SenBeiJia Biological Technology Co., Ltd., Nanjing, China). Biochemical parameters, including albumin, glucose and triglyceride levels, were determined using assay kits (Beijing Leadman Biochemistry Company Limited, Beijing, China) with an automatic biochemical analyzer (Hitachi 7600, Hitachi Ltd., Tokyo, Japan).

### Metabolic profiling and bioanalysis

The frozen liver tissue (100 mg) was thawed at 4 °C and homogenized in 1 mL of precooled methanol/acetonitrile/ddH_2_O (2:2:1, *v*/v/v) with a homogenizer (FastPrep-24™, MP Biomedicals LLC., Santa Ana, California, USA) at 6.0 M/S (20 s each, three times). Then, the mixture was centrifuged for 15 min (14,000×g, 4 °C), and the supernatant was lyophilized under a vacuum and stored at − 80 °C until redissolution in 100 μL of an acetonitrile/water (1:1, v/v) solvent for metabolomics analysis. The untargeted metabolic profiling analysis was conducted using an ultra-performance liquid chromatography (UPLC) system (1290 Infinity LC, Agilent Technologies, Santa Clara, California, USA) coupled to a quadrupole time-of-flight (TOF) mass spectrometer (Triple TOF 6600, AB SCIEX) with electrospray ionization (ESI) in positive and negative ionization modes. For the chromatographic separation, 2 μL of the extracted sample was injected by an autosampler system at 4 °C onto a hydrop interaction liquid chromatography (HILIC) column (ACQUITY UPLC BEH Amide 2.1 mm × 100 mm column, internal diameter 1.7 μm, Waters, Ireland) with a column temperature of 25 °C. In both ESI positive and negative modes, the mobile phase contained an aqueous solution of 25 mM ammonium acetate and 25 mM ammonium hydroxide (A) and acetonitrile (B). The gradient was 85% B and 15% A for 1 min, with a linear reduction to 65% B and 35% A over 11 min, a reduction to 40% B and 60% A over 0.1 min, maintenance for 4 min and an increase to 85% B and 15% A over 0.1 min, with a 5-min re-equilibration period.

For mass spectrometric (MS) detection, the following ESI source conditions were used: ion source gas 1 (Gas1) of 60 psi, ion source gas 2 (Gas2) of 60 psi, curtain gas (CUR) of 30 psi, source temperature of 600 °C, and ion spray voltage floating (ISVF) of ±5500 V. In the MS-only acquisition, the instrument was set to acquire data over the m/z range of 60–1000 Da, and the accumulation time for the TOF MS scan was set to 0.20 s/spectrum. For auto MS/MS acquisition, the instrument was set to acquire data over the m/z range of 25–1000 Da, and the accumulation time for the product ion scan was set to 0.05 s/spectrum. The product ion scan was recorded using information-dependent acquisition (IDA) with the high-sensitivity mode. The parameters were as follows: collision energy (CE): fixed at 35 V ± 15 eV; declustering potential (DP): 60 V (+) and − 60 V (−); exclude isotopes within 4 Da; and the number of candidate ions to monitor per cycle: 10. Quality control (QC) samples were prepared by pooling 10 μL of each sample and were analyzed approximately once every 5 injections to monitor the stability and repeatability of the data produced by the instrument.

### Quantitative RT-PCR

After one aliquot of 100 mg of liver tissue was ground in liquid nitrogen, total RNA was extracted using precooled TriQuick Reagent (Solarbio, Beijing, China) according to the manufacturer’s instructions. The RNA quality was evaluated, and the reverse transcription procedure was performed as described in a previous study [22]. The expression of the target mRNAs was analyzed using real-time PCR (LightCycler® 480, Roche Applied Science, Basel, Switzerland) with a SYBR green-based reaction mixture (SYBR® Premix EX Taq™ II RR 820A, TaKaRa Bio Group, Kusatsu, Japan) containing gene-specific primers (Table 1) according to the manufacturer’s instructions. The primer pairs for all genes were designed using Primer-BLAST software on the website http://www.ncbi.nlm.nih.gov. Amplicon specificity was verified using 1.5% agarose electrophoresis. Relative gene expression levels were normalized to the reference gene *ACTG1* using the 2^-ΔΔCt^ method [23], in which Ct denotes the threshold cycle.

**Table 1:** Gene-specific primers for RT-PCR

Genes	Primer sequences (5′-3′)	Amplicon size (bp)	Accession no.
*ACACA*	F: ATGTGGATGATGGGCTGAA R: GCTTGAACCTGTCGGAAGAG	139	XM_018064168.1
*ACOX1*	F: ACCTGTGAGTTTGTGCCTGA R: TTGGGCTGGAAAGATGCTAC	109	XM_018063769.1
*CPT1α*	F: TCATACTCGCTGGGAACAGA R: TCTCGGAAGGAAACAAATGC	111	XM_018043311.1
*DBP*	F: GATACGGTGGAGGTGCTGAT R: TCCGAGGGTCAAAGGTCTC	91	XM_018062728.1
*G6PC*	F: CCTGCTTCCTGTTCAGTTTCG R: GCAAAGGGCGTCGTGTCAAT	139	XM_005693878.3
*G6PD*	F: ACCTATGGCAACCGATACAAGA R:GTGGAGCAGTGGAGTGAAGAT	144	XM_018044343.1
*INSR*	F: TCAAGGACGGAGTCTTCACC R: TTTCAGCACCTGCTCATTTG	119	XM_018051134.1
*NR1H3*	F: TGCTGATGAAACTGGTGAGC R: TGAAGACACGGAGGAGGAAC	147	NM_001285751.1
*NR3C1*	F: AGAGGGAGAGGGAAATGGAG R: TTGGAATGAGAAGGGTGGTC	121	XM_018050198.1
*PCK1*	F: GCGTTCAACGTCCGATTTCC R: CTCGATGCCGATCTTGGACA	105	XM_005688314.3
*PCK2*	F: TACGTGCTTCCGTTCAGCAT R: TTGGCCCACAGAGTGAAGAC	177	XM_018054616.1
*PRKAA2*	F: TTGATGATGAGGTGGTGGAG R: CCGTGAGAGAGCCAGAGAGT	138	XM_018044652.1
*PRKAB1*	F: CCACCACATCTCCTCCAAGT R: GAGCACCATCACTCCATCCT	135	XM_013970630.2
*PGC1α*	F: CCGAGAATTCATGGAGCAAT R: GATTGTGTGTGGGCCTTCTT	184	XM_018049155.1
*STK11*	F: GGACACCTTCTCTGGCTTCA R: CCCTTCCCGATGTTCTCAA	126	XM_018050463.1
*ACTG1*	F: ATGGCTACTGCTGCGTCGT R: TTGAAGGTGGTCTCGTGGAT	161	XM_018063603.1

### Measurement of the hepatic glycogen content and enzyme activity

Another aliquot of 100 mg of liver tissue was homogenized in 1 mL of precooled sterile saline at 4 °C with a homogenizer (FastPrep-24™, MP Biomedicals LLC., Santa Ana, California, USA) at 6.0 M/S (20 s each, three times); the supernatant was extracted by centrifugation at 1000×*g* for 5 min at 4 °C. The activities of the enzymes hexokinase (HKase), glucose-6-phosphate dehydrogenase (G6PDHase), glucose 6-phosphatase (G6Pase), and phosphoenolpyruvate carboxykinase (PEPCKase) were determined using commercially available assay kits (Solarbio, Beijing Solarbio Life Sciences Ltd., Beijing, China) according to the manufacturer’s instructions. The results are displayed as U/g (fresh tissue). The glycogen content was determined using a commercially available kit (Nanjing Jiancheng Bioengineering Research Institute, Nanjing, China), and the results are presented as mg/g (fresh tissue).

### Bioinformatics and statistical analysis

The raw MS data (wiff.scan files) were converted to MzXML files using the ProteoWizard MSConvert tool before importing the data into XCMS software for peak detection and alignment. Collection of Algorithms of Metabolite profile Annotation (CAMERA) was used to annotate isotopes and adducts. In the extracted ion features, only variables with greater than 50% nonzero measurement values in at least one group were retained. After normalizing to the total peak intensity, the processed data were imported into SIMCA-P (version 14.1, Umetrics, Umea, Sweden) and Pareto-scaled, and principal component analysis (PCA) and orthogonal partial least squares discriminant analysis (OPLS-DA) were conducted. After seven-fold cross-validation and response permutation testing were performed to evaluate the robustness of the OPLS-DA model, the differentiated ion peaks with variable importance for the projection (VIP) > 1, a correlation coefficient between the X variables and a predictive score of the predictive component 1 [*p* (corr)] > 0.6 were selected. Identification of the compounds to which the differentiated ion peaks were attributed was performed by comparing the accuracy of the m/z values (< 25 ppm) and MS/MS spectra with an in-house database established with available authentic standards. Then, the significantly different (*P* < 0.05) and potentially different (0.05 < *P* < 0.10) metabolites were analyzed using an independent *t*-test, subjected to a free online Kyoto Encyclopedia of Genes and Genomes (KEGG) pathway enrichment program (MetaboAnalyst 3.0, http://www.metaboanalyst.ca/), and analyzed using Fisher’s exact test with significance set to *P* < 0.05.

Phenotypic, physiological and mRNA data were analyzed using a mixed model in SPSS 19.0 statistical software (IBM SPSS Inc., 2010). The variables of BW and liver weight of the dams at 100 d of gestation were analyzed with treatment as a fixed factor and the initial BW of the dams at 45 d of gestation as a covariate; other characteristics of the dams were analyzed with treatment as a fixed factor. The nonmetabolomic data from the fetuses and kids were grouped by sex and analyzed with treatment as a fixed factor and the initial BW of the dams as a covariate. All data are presented as the estimated marginal means and standard error of the means (EM means ± SEM), and significance was considered at *P* < 0.05, with a significant trend defined at 0.05 ≤ *P* ≤ 0.10.

## Results

### Physical and blood biochemical parameters

Compared with the C dams, the R dams had a lower BW (*P* = 0.008), total weight (*P* = 0.001) and relative liver weight (*P* = 0.025) (Table 2). Undernutrition increased (*P* = 0.005) the plasma glucagon concentration in the dams, but the albumin, glucose and triglyceride concentrations and other hormones levels were not affected.

**Table 2:** Physical and blood biochemical parameters of the pregnant dams (*n* = 6)

	C^*a*^	R^*a*^	SEM^*b*^	*P* value
Body weight, kg	38.8	33.2	1.17	0.008
Liver weight, g	718.0	534.4	26.34	0.001
Liver weight relative to BW, g/100 g	1.95	1.72	0.061	0.025
Albumin, g/L	38.53	36.98	1.296	0.42
Glucose, mmol/L	3.48	2.84	0.542	0.43
Triglycerides, mmol/L	0.44	0.36	0.059	0.34
Growth hormone, μg/L	16.97	14.37	1.993	0.38
IGF-I^*c*^, μg/L	192.19	162.44	35.743	0.57
Insulin, μIU/mL	15.48	11.67	2.135	0.24
Glucagon, ng/L	25.88	52.25	5.002	0.005
Cortisol, μg/L	130.40	137.98	18.205	0.77

^a^The C group of dams was fed 100% of their nutrient requirements as outlined in the Chinese Meat Goat Requirements (2004). The R group of dams was fed 60% of the intake of the C group from 55 to 100 d of gestation and then realimented to 100%. The dams and fetuses were harvested at 100 d of gestation; the kids were weaned at 60 d and harvested 90 d after birth

^b^*SEM* pooled standard error of the mean

^c^Insulin-like growth factor 1

The effect of maternal undernutrition on the physical and blood biochemical parameters of the offspring differed based on age and sex (Table 3). In the male offspring, the BW of the R fetuses tended to be lower (*P* = 0.060), and the concentrations of triglyceride and GH in the R fetuses were decreased (*P* < 0.05); the albumin concentration in the R kids tended to be higher (*P* = 0.062), and GH and cortisol concentrations in the R kids were increased (*P* < 0.05). In the female offspring, no effect was observed in the physical and blood biochemical parameters in the fetuses or kids.

**Table 3:** Physical and blood biochemical parameters of the fetuses at 100 d of gestation and postnatal kids at 90 d

	Fetuses				Kids			
	C^*a*^	R^*a*^	SEM^*b*^	*P* value	C	R	SEM	*P* value
Males, n	7	6			3	4		
Body weight, kg	664.4	529.7	44.05	0.060	9.8	7.8	0.93	0.19
Liver weight, g	40.9	32.3	3.51	0.12	198.7	145.9	20.48	0.14
Liver weight relative to BW, g/100 g	6.14	6.09	0.209	0.88	2.01	2.01	0.073	0.97
Albumin, g/L	19.22	18.20	0.573	0.28	40.12	43.94	1.046	0.062
Glucose, mmol/L	2.46	1.09	0.980	0.38	3.69	2.56	0.569	0.23
Triglyceride, mmol/L	0.72	0.50	0.047	0.024	0.56	0.68	0.080	0.34
Growth hormone, μg/L	92.13	78.22	2.631	0.008	11.13	14.28	0.683	0.031
IGF-I^*c*^, μg/L	48.17	51.43	6.277	0.73	131.26	83.91	35.114	0.40
IGF-II^*c*^, μg/L	21.49	25.49	2.111	0.22	ND^*d*^	ND		
Insulin, μIU/mL	7.34	5.52	1.651	0.47	8.64	8.91	0.353	0.63
Glucagon, ng/L	32.83	32.77	1.283	0.98	31.50	31.57	6.927	0.99
Cortisol, μg/L	10.06	13.04	2.301	0.39	78.83	136.69	5.149	0.001
Females, n	3	4			5	4		
Body weight, kg	569.2	624.7	40.57	0.39	8.8	7.2	0.62	0.11
Liver weight, g	37.5	37.6	2.36	0.98	173.8	138.3	17.70	0.21
Liver weight relative to BW, %	6.56	6.04	0.186	0.12	2.01	2.02	0.101	0.99
Albumin, g/L	–	–			42.85	39.79	1.951	0.31
Glucose, mmol/L	–	–			2.98	2.65	0.326	0.50
Triglyceride, mmol/L	–	–			0.56	0.51	0.117	0.79
Growth hormone, μg/L	82.80	78.95	6.915	0.78	17.49	17.77	2.714	0.94
IGF-I, μg/L	55.66	75.49	16.595	0.53	100.07	87.13	15.976	0.59
IGF-II, μg/L	20.60	26.23	1.383	0.18	ND	ND		
Insulin, μIU/mL	6.81	5.86	0.680	0.44	8.58	8.86	0.627	0.76
Glucagon, ng/L	34.58	33.29	0.906	0.43	34.79	33.14	5.074	0.83
Cortisol, μg/L	18.91	18.63	3.167	0.96	108.52	138.97	16.311	0.24

^a^The C group of dams was fed 100% of their nutrient requirements as outlined in the Chinese Meat Goat Requirements (2004). The R group of dams was fed 60% of the intake of the C group from 55 to 100 d of gestation and then realimented to 100%. The dams and fetuses were harvested at 100 d of gestation; the kids were weaned at 60 d and harvested 90 d after birth

^b^*SEM* pooled standard error of the mean

^c^Insulin-like growth factor 1 or 2

^d^*ND* not detected

### Global metabolic profiling of the liver

**Fig. 2:**
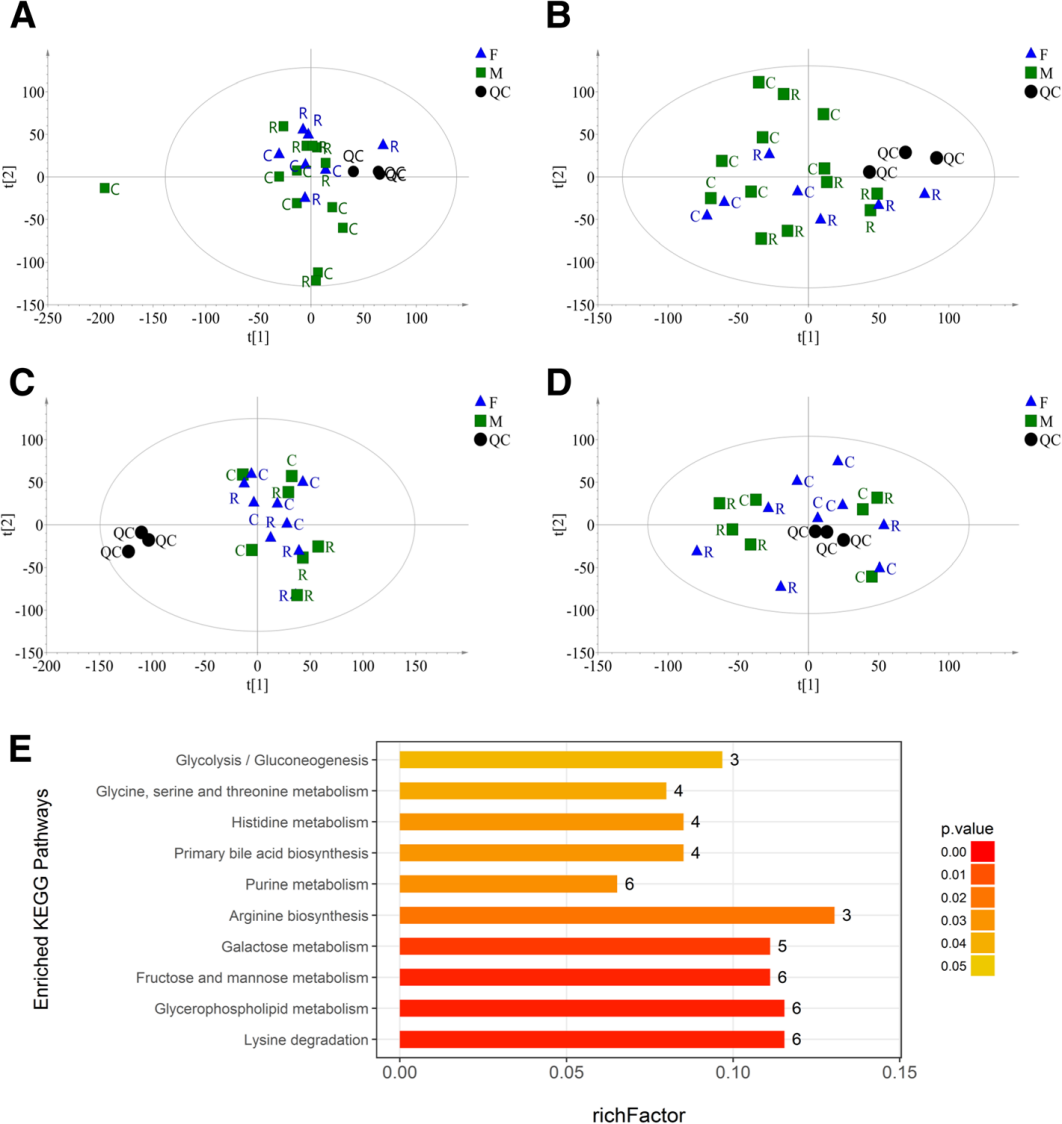
Metabolite profiling analysis of offspring livers. **a**-**d** Scatter plots of the PCA-X model based on all identified metabolite features of liver samples from the fetuses (**a**, positive mode; **b**, negative mode) and kids (**c**, positive mode; **d**, negative mode) in the control (C) or restricted (R) group, including the quality control (QC) samples. **e** The enriched KEGG pathways of the differential metabolites in the fetal liver. richFactor = the ratio of the number of differential metabolites to total metabolites in each pathway

Based on the extracted ion peaks, the scatter plots of all samples, including the quality control (QC) samples (Fig. 2a-d), indicated the precision and repeatability of the untargeted UPLC-MS detection. The metabolic profile was indistinguishable between the male and female fetuses (Fig. 2a and b**)** and kids (Fig. 2c and d**)** in both the positive and negative modes**;** thus**,** we did not regroup and analyze the metabolomics data by sex. According to Hotelling’s T2 ellipse in the PCA-X model (Fig. 2a), one outlier was eliminated from the C and R groups of fetuses. In the fetuses, 8451 and 8231 ion peaks were extracted in the positive and negative modes, respectively. The OPLS-DA analysis revealed a clear distinction between the two groups in both positive [cumulative variation modeled in the component in the Y matrix (R^2^Y_cum_) = 0.999, cumulative estimate of the predictive ability of the model (Q^2^_cum_) = 0.587)] and negative modes (R^2^Y_cum_ = 0.990, Q^2^_cum_ = 0.577), which was validated by the permutation analysis (positive: Q^2^_intercept_ = − 0.034; negative: Q^2^_intercept_ = − 0.209). One hundred eighteen metabolites (57 in positive mode and 61 in negative mode) with significant differences (*P* < 0.05) or potential differences (0.05 ≤ *P* < 0.10) were identified. The KEGG pathway enrichment analysis shown in Fig. 2e revealed 10 pathways that were affected by maternal undernutrition, including amino acid metabolism, monosaccharide metabolism, phospholipid metabolism, and purine and bile acid metabolism. Among these ten pathways, 65.96% (31 of 47) of the differential metabolites were upregulated, as shown in Table 4.

**Table 4:** Differential metabolites from fetal livers in the top ten enriched KEGG pathways

Metabolite^*a*^	m/z	rt(s)	VIP	Fold change	*P* value
Lysine degradation					
N6-Acetyl-L-lysine	189.12	653.67	1.85	1.30	0.022
L-Lysine	147.11	984.76	4.86	1.19	< 0.000
L-Pipecolic acid	130.09	984.75	3.83	1.19	< 0.000
Glutaric acid	131.04	491.25	1.39	0.49	0.046
DL-2-Aminoadipic acid	162.08	697.23	1.37	1.68	0.066
Glycine	74.03	704.95	1.19	1.30	0.002
Glycerophospholipid metabolism					
Glycerol 3-phosphate	171.01	816.37	5.92	1.18	0.053
Glycerophosphocholine	258.11	763.17	1.76	0.65	0.023
Phosphorylcholine	242.08	732.23	4.08	0.73	0.045
Acetylcholine	146.12	709.06	5.58	0.82	0.040
O-Phosphoethanolamine	140.01	864.35	2.45	1.12	0.082
sn-Glycerol 3-phosphoethanolamine	216.06	740.82	7.13	0.69	0.038
Fructose and mannose metabolism					
Alpha-D-Glucose	179.06	557.54	9.79	1.44	0.012
D-Sorbitol	181.07	556.12	13.69	0.72	0.014
D-Mannitol	183.09	551.07	5.18	0.71	0.046
D-Mannose	198.10	551.23	3.52	1.40	0.032
Beta-D-Fructose 6-phosphate	259.02	814.70	1.60	0.77	0.018
DL-lactate	89.03	556.73	1.15	1.24	0.088
Galactose metabolism					
Alpha-D-Glucose	179.06	557.54	9.79	1.44	0.012
D-Sorbitol	181.07	556.12	13.69	0.72	0.014
D-Mannose	198.10	551.23	3.52	1.40	0.032
Glycerol	151.06	187.25	1.08	1.06	0.081
D-Tagatose	179.06	370.37	1.15	0.65	0.061
Arginine biosynthesis					
L-Glutamine	145.06	699.84	5.05	1.08	0.037
L-Pyroglutamic acid	130.05	559.43	2.25	1.25	0.007
L-Citrulline	176.10	731.72	3.45	1.42	0.007
Purine metabolism					
L-Glutamine	145.06	699.84	5.05	1.08	0.037
Inosine	269.09	306.89	1.26	0.41	0.031
Hypoxanthine	135.03	304.52	5.86	1.06	0.084
Adenosine	250.09	186.16	1.65	0.77	0.071
Adenosine 5′-diphosphate (ADP)	426.02	870.44	1.04	1.19	0.072
Glycine	74.03	704.95	1.19	1.30	0.002
Primary bile acid biosynthesis					
Taurochenodeoxycholate	498.29	251.31	10.41	1.48	0.047
Glycine	74.03	704.95	1.19	1.30	0.002
Taurocholate	516.30	381.79	1.89	0.67	0.067
Glycocholic acid	483.34	465.40	1.68	0.45	0.022
Histidine metabolism					
1-Methylhistidine	170.09	691.63	2.94	1.34	0.069
L-Carnosine	227.11	791.19	3.32	0.79	0.012
L-Histidine	156.08	706.27	3.82	1.26	0.022
1-Methylhistamine	126.10	624.34	3.19	2.84	0.033
Glycine, serine and threonine metabolism					
DL-Serine	104.04	704.80	6.00	1.29	0.001
Glycine	74.03	704.95	1.19	1.30	0.002
L-Threonine	120.07	654.22	2.79	1.14	0.007
Glycolysis/Gluconeogenesis					
alpha-D-Glucose	179.06	557.54	9.79	1.44	0.012
D-Glucose 6-phosphate	261.04	895.44	4.43	0.67	0.065
DL-lactate	89.03	556.73	1.15	1.24	0.088

^a^Differential metabolites were identified based on the variable importance for a projection (VIP) value > 1, a correlation coefficient between the X variables and a predictive score of predictive component 1 [*p* (corr)] > 0.6 in the OPLS-DA model and an independent t-test with a significant difference (*P* < 0.05) and a potential difference (0.05 < *P* < 0.10). If one differential metabolite was detected in both positive and negative modes, the parameters from positive mode are presented

Regarding the hepatic metabolic profile of the kids, 18,654 and 14,784 ion peaks were extracted in positive and negative modes, respectively. No abnormal sample was found, but the eigenvalues from the OPLS-DA models did not indicate a clear separation between the groups in either the positive (R^2^Y_cum_ = 1.0, Q^2^_cum_ = 0.447, Q^2^_intercept_ = 0.333) or negative (R^2^Y_cum_ = 0.994, Q^2^_cum_ = 0.241, Q^2^_intercept_ = 0.052) ion mode.

### Energy metabolism-related gene expression

**Fig. 3:**
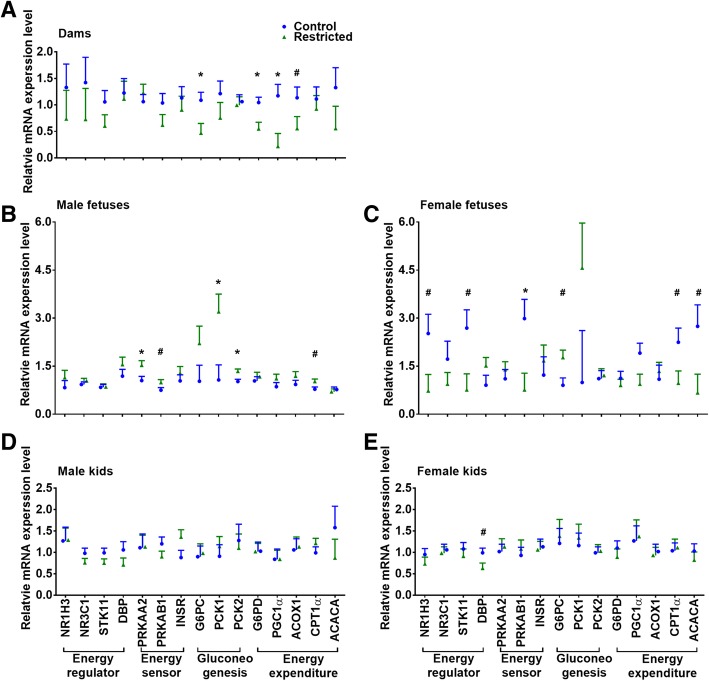
Relative mRNA expression of genes related to energy metabolism in the livers of dams (**a**), fetuses (**b** and **c**) and kids (**d** and **e**). Data were analyzed with a mixed model, and the results are presented as the mean ± SE; *P < 0.05 and # 0.05 ≤ *P* < 0.10

The mRNA expression of the energy metabolism-related genes in the liver tissue is presented in Fig. 3. In the dams (Fig. 3a), the expression of the *ACOX1* (*P* = 0.097), *G6PC* (*P* = 0.030) and *G6PD* (*P* = 0.015) mRNAs was reduced by approximately 50% in the R group compared to that in the C group; in particular, the *PGC1α* mRNA level was decreased (*P* = 0.022) by 81% in the R group. In the male fetuses (Fig. 3b), compared with the C group, the *CPT1α* and *PRKAB1* mRNA levels in the R group tended to be increased (*P* < 0.10), while the levels of the *PRKAA2*, *PCK1* and *PCK2* mRNAs were increased (*P* < 0.05) by 130 to 340%. In the female fetuses (Fig. 3c), the *PRKAB1* mRNA level in the R group was decreased (*P* = 0.049), and the *ACACA*, *CPT1α*, *NR1H3*, and *STK11* mRNA expression in the R group tended to be lower (*P* < 0.10) than that in the C group, while the *G6PC* mRNA level in the R fetuses tended to be higher (*P* = 0.082) than that in the C fetuses. However, the expression of all detected genes in the male kids were unaffected by maternal undernutrition (Fig. 3d), and only the *DBP* mRNA level in the female R kids tended to be lower (*P* = 0.073) than that in the C kids (Fig. 3e).

### Hepatic glycogen content and glucose metabolism-related enzyme activities

**Fig. 4:**
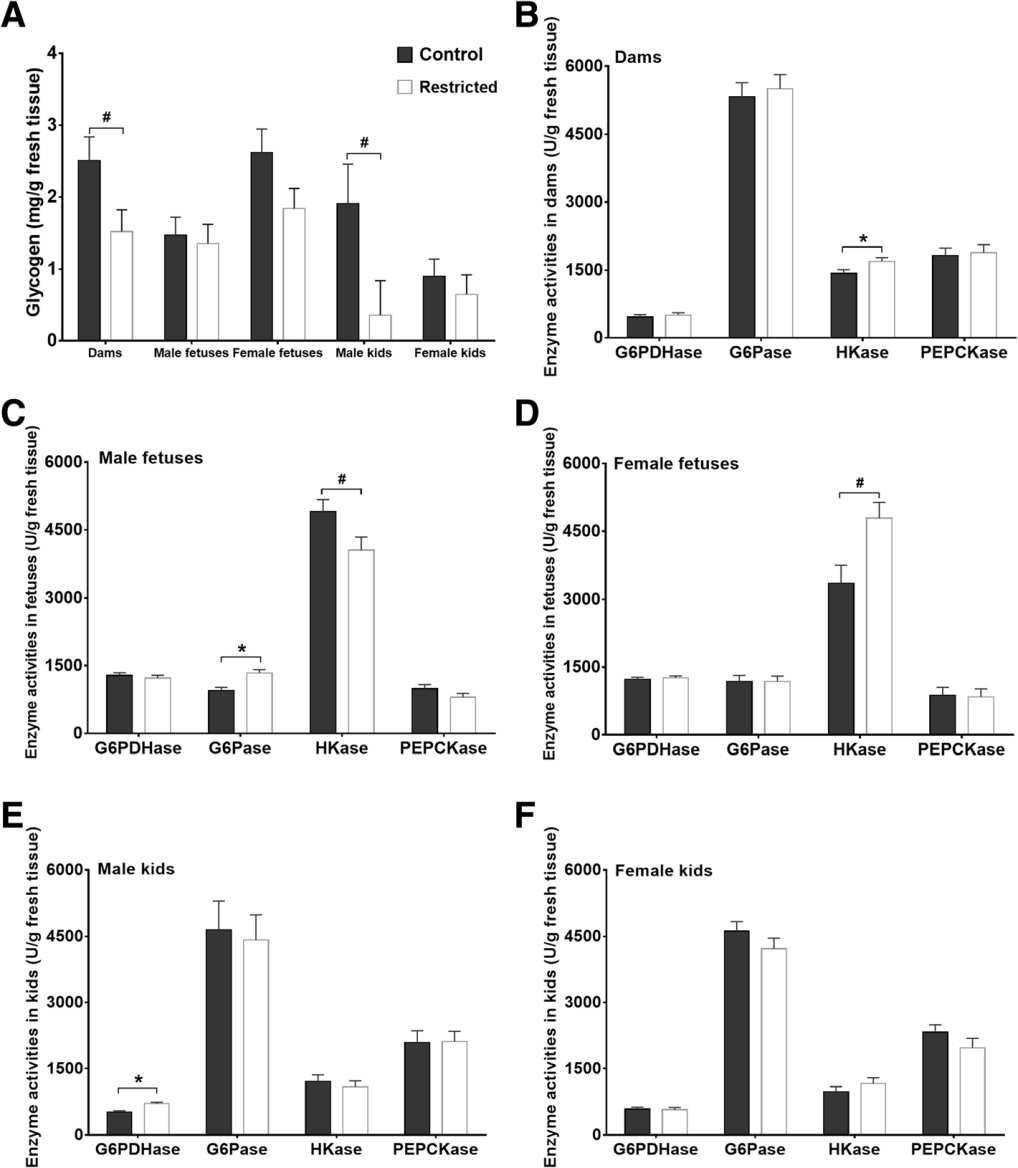
Hepatic glycogen contents and enzyme activities. **a** The glycogen contents in dams, fetuses and kids. **b**-**f** The enzyme activities in the dams and offspring. Data were analyzed with a mixed model, and the results are presented as the mean ± SE; *P < 0.05 and # 0.05 ≤ *P* < 0.10. G6PDHase = glucose-6-phosphate dehydrogenase; G6Pase = glucose 6-phosphatase; HKase = hexokinase; PEPCKase = phosphoenolpyruvate carboxykinase

The hepatic glycogen content and activities of enzymes related to glucose metabolism are depicted in Fig. 4. Compared with the C dams, the glycogen content tended to be lower (*P* = 0.055) in the R dams (Fig. 4a), but the activity of HKase was increased (*P* = 0.042) in the R dams (Fig. 4b). Compared with the C fetuses, the activity of G6Pase in the R males was increased (*P* = 0.003), and the HKase activity tended to be decreased (*P* = 0.062, Fig. 4c), while the HKase activity tended to be higher (*P* = 0.055) in the R females than that in the C females (Fig. 4d). However, in the kids, the glycogen content in the R males tended to be lower (*P* = 0.10) than that in the C males, and the G6PDHase level in the R males was higher than that in the C males (*P* = 0.01, Fig. 4e). No differences were observed in the glycogen content and glucose metabolism-related enzyme activities between the female C and R kids (Fig. 4f).

## Discussion

To the best of our knowledge, this report successfully describes the successive effects of maternal undernutrition during midgestation on hepatic energy metabolism in fetuses and juveniles. Recently, increasing studies have shown that the impact of maternal malnutrition on offspring is sex-specific. Our results further confirm that the effect of maternal undernutrition during midgestation on the physical phenotype, blood metabolites, hormones, and hepatic metabolism of prenatal and postnatal offspring differed in a sex-specific manner.

In this study, the 40% nutrient deficiency did not alter the BW, liver weight, or blood metabolite levels in the female fetuses, but the BW and triglyceride and GH levels in the male fetuses were reduced by varying degrees. Similarly, in the postnatal kids, all physical and circulating blood metabolites measured were unaffected in the females, but the concentrations of albumin, GH and cortisol in the restricted males were increased. Previously, our research team found that maternal 40% energy restriction during late gestation resulted in a higher relative liver weight [20] and lower insulin concentration [21] in 7-week-old male kids. The different outcomes from previous studies may be due to the pregnancy stage (mid vs. late gestation) and the type of nutritional constraints. A global restriction of feed intake was implemented in this study instead of energy or protein level limitation in the previous studies. The higher level of glucagon observed in the restricted dams was undoubtedly induced by the lower feed intake following the nutrient restriction in this study. The reduction in BW and liver weight in the restricted dams is suggestive of the mobilization of the body reserve, which partially explains the intact physical outcome in the female offspring in the present study, but male offspring were more intensely affected. The sex disparity is likely related to sex differences in nutrient sensing [24] and metabolism responses to undernutrition [25, 26], and female estrogen can perform a protective action during early life programming via IGF-1 [27] or NR1H3 [28] as observed in adult mice, which needs further confirmation in goats. In fetal metabolic programming following exposure to maternal undernutrition during midgestation, hormones belonging to the GH-hepatic IGF-1/IGF-II endocrine axis are key regulatory signals in mammals, such as pigs [29] and sheep [30]. The GH-IGF-1 axis is also a vital factor for postnatal catch-up growth [31]. In this study, maternal undernutrition also induced the fetal programming of GH, and this effect persisted in the kids after birth. Furthermore, the disturbed GH secretion pattern is retroacted to the development of the hypothalamus and pituitary gland [32], and maternal undernutrition programs the downstream hormone response to stress in offspring [33]. We propose that the increased cortisol concentration in the male kids was also due to this pathway of action and that the higher albumin content was resultantly generated to act as a transport protein for steroid hormones of cortisol. Similarly, plasma cortisol was increased in lambs after maternal undernutrition between 28 and 78 d of gestation [34]. This thrifty phenotype and programmed endocrine secretion pattern generally increase the risk of metabolic disorders later in life after long-term overnutrition [5, 6].

The metabolic profile of offspring at different developmental stages helps further reveal the mechanism of metabolic programming caused by maternal undernutrition. Separately examining the sex differences in the metabolic profiles could facilitate an understanding of the above sex-specific outcomes. However, based on the scatter plots (Fig. 2a-d), the metabolic profiles were not obviously differentiated between the females and males. The limited number of samples (e.g., three female restricted fetuses) may have weakened the persuasiveness of the results; thus, we cannot affirm that no sex-specific difference exists in the metabolic profiles of offspring. Based on the available data, maternal undernutrition alters the metabolic profile of the liver during the fetal period but not the postnatal period. As a pivotal organ for glucose, amino acid, lipid, and bile acid metabolism, liver metabolic pathways correlated with these nutrients in the restricted fetuses were extensively altered (Table 3). However, among the identified differential metabolites, most were gluconeogenic substrates, including glucogenic amino acids (such as lysine, glycine, histidine, glutamine, serine and threonine), lactate and glycerol, which were increased in the restricted fetuses. Therefore, in the present study, we mainly focused on glycolysis and gluconeogenesis pathways and further examined the related genes expression and enzymes involved.

Due to the irreplaceable role of glucose in fetal energy supply, mammals have developed an effective mechanism to meet the glucose demand through gluconeogenesis. The downregulated *G6PC*, *G6PD*, *PGC1α* and *ACOX1* mRNA levels and the increased hexokinase activity in the restricted pregnant dams in the current study imply the globally reduced energy expenditure and activation of a glucose-sparing pathway in the mothers. However, the effect of maternal undernutrition on the mRNA expression and enzyme activities related to glucose metabolism in the offspring was also sex-specific and varied with the developmental stage. The levels of the gluconeogenesis-related genes *PCK1* and *PCK2* were increased by 299 and 131%, respectively, in the restricted male fetuses. The expression of these genes is subtly manipulated in gluconeogenesis; in particular, *PCK* is synergistically regulated by insulin, glucocorticoids, glucagon, and cAMP via the phosphorylation of Ser_133_ located in a beta sheet of the cAMP response element-binding protein [35]. Accordingly, the expression of *PRKAA2* and *PRKAB1* mRNA in the restricted male fetuses was increased by 146 and 132%, respectively, in the current study. PRKAA2 and PRKAB1 are the catalytic and regulatory subunits of AMP-activated protein kinase (AMPK), which monitors the cellular energy status by detecting the AMP/ATP ratio [36].The activity of the G6Pase enzyme in the restricted male fetuses was increased, and the HKase activity was decreased in this study. HKase is responsible for the first step in glycolysis to phosphorylate glucose, while G6Pase catalyzes the final step of gluconeogenesis to transform glucose 6-phosphate into glucose for release into circulation. These changes along with the increased *CPT1α* mRNA level indicate a deficiency in glucose and the initiation of gluconeogenesis in the restricted male kids. In contrast to the effect on the male fetuses, the HKase activity and *G6PC* mRNA expression were increased in the restricted female fetuses. The expression level of genes involved in lipid metabolism (*ACACA*, *CPT1α* and *NR1H3*) and energy sensing (*PKB1* and *STK11*) was decreased in the restricted female fetuses. Moreover, notably, while differences in the hepatic glycogen content (2.62 vs. 1.47 mg/g), HKase activity (3345 vs. 4901 U/g), and gene expression levels (the levels of *ACACA*, *CPT1α*, *NR1H3*, *PKB1* and *STK11* in the control females were twice those in the control males) were observed between the control female and control male fetuses, these variables were similar between the restricted female and restricted male kids. Due to the sexual dimorphism of energy sensing and glucose metabolism in the liver (as summarized by [37]), we infer that maternal undernutrition attenuates this metabolic dimorphism via sex-specific metabolic adaptation to maintain glucose homeostasis. The simultaneous alteration in HKase activity in both genders, and *PCK*s mRNA and G6Pase activity in male fetuses or *G6PC* mRNA in females explains the increase in alpha-D-glucose and the reduction in D-glucose 6-phosphate in the hepatic metabolites of the restricted fetuses. In addition, a close relationship exists between bile acids and gluconeogenic genes. Taurocholate and taurochenodeoxycholate regulate glucose synthesis from lactate and pyruvate by mediating *PCK* and *G6PC* mRNA expression [38, 39]; therefore, the lower taurocholate level and higher taurochenodeoxycholate and lactate levels in the livers of the restricted fetuses partially contributed to gluconeogenesis. Similarly, increased lactate was also reported in the livers of growth-restricted fetal sheep [40], and the glucogenic pathway is initiated prematurely in the ovine fetus under hypoglycemia [41]. Based on these results, we suggest that 40% maternal undernutrition triggers the early activation of the gluconeogenesis pathway in fetuses in a sex-specific manner.

After realimentation, maternal undernutrition did not affect the metabolic profile of the liver in the postnatal kids. The hepatic glycogen content and activities of energy metabolism-related enzymes in the female kids were unaffected, but the G6PDHase activity was increased, and the glycogen content was decreased in the restricted male kids, which may be explained by the high cortisol level in the restricted male kids. Cortisol exerts permissive effects on normal activation of hepatic gluconeogenesis and glycogenolysis [8], while G6PDHase catalyzes the production of NADPH, which acts as the cofactor for 11 beta-hydroxysteroid dehydrogenase type 1 to increase the regeneration of active glucocorticoids [42, 43]. In addition, maternal undernutrition scarcely affected the tested mRNA expression of hepatic glucose metabolism genes in the female (except for *DBP* mRNA) or male kids in this study. In contrast, similar studies have found that maternal undernutrition during midgestation upregulates the hepatic expression of *PCK* and *glucocorticoid receptor* genes in male lambs [16], and increases the *PCK* and downregulates the *peroxisome proliferator-activated receptor-γ* [7] in female sheep. The main reason is that metabolic abnormalities are often found in adulthood after long-term exposure to an obesogenous environment, such as feeding commercial pelleted concentrate mixtures ad libitum for nine months [16] or 11 weeks in aged offspring [7] in these studies. During the juvenile stage, in this study, the kids were not exposed to an overnutrition environment; thus, an abnormal metabolism was not induced. Next, research investigating the sexual dimorphism of fetal programming in liver metabolism following a nutritional challenge needs to be addressed in goats.

## Conclusions

The thrifty phenotype hypothesis suggests that intrauterine undernutrition programs the fetal metabolic trajectory. In this study, 40% maternal undernutrition changed the metabolic profile, altered the sex-specific expression of mRNAs involved in the activation of gluconeogenesis, and affected the activities of HKase and G6Pase in the livers of the fetuses, but these signs of altered metabolic phenotypes were almost eliminated in the juvenile animals after realimentation. However, the changes in the circulating hormone levels in the male offspring provide evidence of metabolic programming, which may be included in surveillance before clinical signs appear. Moreover, other altered metabolic pathways in the offspring, including amino acid, lipid and bile acid metabolism, need to be further addressed.

## Abbreviations

*ACACA*: Acetyl-CoA carboxylase alpha
*ACOX1*: Acyl-CoA oxidase 1
*ACTG1*: Actin gamma 1
*CPT1α*: Carnitine palmitoyltransferase 1A
*DBP*: D-box binding PAR bZIP transcription factor
*G6PC*: Glucose-6-phosphatase catalytic subunit
*G6PD*: Glucose-6-phosphate dehydrogenase
*INSR*: Insulin receptor
*NR1H3*: Nuclear receptor subfamily 1 group H member 3
*NR3C1*: Nuclear receptor subfamily 3 group C member 1
*PCK1*: Phosphoenolpyruvate carboxykinase 1
*PCK2*: Phosphoenolpyruvate carboxykinase 2, mitochondrial
*PGC1α*: Peroxisome proliferator-activated receptor gamma coactivator 1 alpha
*PRKAA2*: Protein kinase AMP-activated catalytic subunit alpha 2
*PRKAB1*: Protein kinase AMP-activated noncatalytic subunit beta 1
*STK11*: Serine-threonine kinase 11
